# Clinical features, socioeconomic status, management, short and long-term outcomes of patients with acute myocardial infarction: Phase I results of PEACE MENA registry

**DOI:** 10.1371/journal.pone.0296056

**Published:** 2024-01-11

**Authors:** Ayman Al Saleh, Amal Jamee, Kadhim Sulaiman, Mohamed Sobhy, Habib Gamra, Fahad Alkindi, Salim Benkhedda, Ahmed Al-Motarreb, Mohammad I. Amin, Wael Almahmeed, Ayman Hammoudeh, Hadi Skouri, Hasan A. Farhan, Mohammad Al Jarallah, Nadia Fellat, Prashanth Panduranga, Bayan K. Alnajm, Magdy Abdelhamid, Rafik Refaat, Hassen Amor, Salma Messaous, Hosameldin S. Ahmed, Ahcene Chibane, Azzouz AbdulMalek, Nora K. Alsagheer, Sobhi Dada, Zaki Mokhtar, Muhammad Ali, Anhar Ullah, Hanan AlBackr, Khalid F. Alhabib

**Affiliations:** 1 Department of Cardiac Sciences, King Fahad Cardiac Center, College of Medicine, King Saud Medical City, King Saud University, Riyadh, Saudi Arabia; 2 Nassar Medical Complex Hospital, Ministry of Health, Khan Younes, Gaza Strip, Palestine; 3 Al-Quds Hospital, Gaza, Palestine; 4 National Heart Center, Royal Hospital, Muscat, Oman; 5 International Cardiac Center (ICC), Alexandria, Egypt; 6 Research Laboratory LR, Fattouma Bourguiba University Hospital, University of Monastir, Monastir, Tunisia; 7 Heart Hospital, Hamad Medical Corporation, Doha, Qatar; 8 Cardiology Department, Mustapha Hospital, COCRG Laboratory University Benyoucef Benkhedda, Algiers, Algeria; 9 Faculty of Medicine, Sana’a University, Sana’a, Yemen; 10 Mohamed Bin Khalifa Cardiac Centre, Awali, Bahrain; 11 Heart and Vascular Institute, Cleveland Clinic Abu Dhabi, United Arab Emirate; 12 Cardiology Department, Istishari Hospital, Amman, Jordan; 13 Cardiology Division, Internal Medicine Department at American University of Beirut Medical Center, Beirut, Lebanon; 14 Iraqi Board for Medical Specializations, Scientific Council of Cardiology. Baghdad Heart Center, Medical City, Baghdad, Iraq; 15 Sabah Al Ahmed Cardia Center, Kuwait City, Kuwait; 16 IBN SINA University Hospital, Rabat, Morocco; 17 Al Nahdha Hospital, Muscat, Oman; 18 Faculty of Medicine, Kasr Al Ainy Hospital, Cairo University, Giza Governorate, Egypt; 19 Taher Sfar University Hospital, Mahdia, Tunisia; 20 Internal Medicine and Cardiology Department, CHU Douéra, Algiers, University Saad Dahlab, Blida, Algeria; 21 Althawra Modern General Hospital, Sana’a, Yemen; 22 Hammoud University Medical Center, Saida, Lebanon; 23 King Saud Hospital, Unizah, Qaseem, Saudi Arabia; 24 Saudi Heart Association, Riyadh, Saudi Arabia; 25 National Heart and Lung Institute, Imperial College London, London, United Kingdom; Tabba Heart Institute, PAKISTAN

## Abstract

**Background:**

The Program for the Evaluation and Management of Cardiac Events in the Middle East and North Africa (PEACE MENA) is a prospective registry program in Arabian countries that involves in patients with acute myocardial infarction (AMI) or acute heart failure (AHF).

**Methods:**

This prospective, multi-center, multi-country study is the first report of the baseline characteristics and outcomes of inpatients with AMI who were enrolled during the first 14-month recruitment phase. We report the clinical characteristics, socioeconomic, educational levels, and management, in-hospital, one month and one-year outcomes.

**Results:**

Between April 2019 and June 2020, 1377 patients with AMI were enrolled (79.1% males) from 16 Arabian countries. The mean age (± SD) was 58 ± 12 years. Almost half of the population had a net income < $500/month, and 40% had limited education. Nearly half of the cohort had a history of diabetes mellitus, hypertension, or hypercholesterolemia; 53% had STEMI, and almost half (49.7%) underwent a primary percutaneous intervention (PCI) (lowest 4.5% and highest 100%). Thrombolytics were used by 36.2%. (Lowest 6.45% and highest (90.9%). No reperfusion occurred in 13.8% of patients (lowest was 0% and highest 72.7%).Primary PCI was performed less frequently in the lower income group vs. high income group (26.3% vs. 54.7%; P<0.001). Recurrent ischemia occurred more frequently in the low-income group (10.9% vs. 7%; P = 0.018). Re-admission occurred in 9% at 1 month and 30% at 1 year, whereas 1-month mortality was 0.7% and 1-year mortality 4.7%.

**Conclusion:**

In the MENA region, patients with AMI present at a young age and have a high burden of cardiac risk factors. Most of the patients in the registry have a low income and low educational status. There is heterogeneity among key performance indicators of AMI management among various Arabian countries.

## Introduction

Cardiovascular disease (CVD) is the leading cause of mortality and morbidity worldwide. The presence of coronary artery disease risk factors has been clearly associated with an increased risk of cardiovascular events (CVEs) and mortality. Less is understood about the relationship between socioeconomic status (SES) and CVD.

A close relationship has been demonstrated between a higher prevalence of cardiovascular risk factors (CVRs) and lower SES and the development of complications and early death [1]. This relationship has changed over time, with cardiovascular burden in the early 20th century being greater among people with higher SES due to the higher prevalence of smoking and sedentary and unhealthy lifestyles. This shift has occurred due to the development of various treatment modalities and cardiovascular prevention having a greater impact on people with lower SES [2]. This shift has been demonstrated among lower socioeconomic groups in both low- and middle-income countries [3, 4].

The Arabian countries in the Middle East and North Africa (MENA) region have a population of ~350 million, which represents approximately 6% of the world’s population. These people share some common cultural, traditional, environmental, and lifestyle factors, as well as a few gene clusters in their genomes. However, they have diverse health care systems with extremely variable national economies, ranging from low-income to high-income countries.

Here, we present the clinical characteristics, management, in-hospital outcomes, 30-day and one-year readmission and cardiac mortality rates of patients admitted with acute myocardial infarction (AMI) in phase 1 of the Program for the Evaluation and Management of Cardiac Events in the MENA region (PEACE MENA) registry.

Phase 1 was a 14-month recruitment phase that included consecutive inpatients presenting with AMI, with longitudinal follow-ups at 30 days and 1 year. Patients were recruited from 32 hospitals in 16 Arabian countries with and without catheterization laboratories (Cath and non-Cath, respectively) to reflect real-life health care practices.

The objectives were to describe the demographic characteristics, SES (including monthly household income, health care coverage, and education levels), clinical presentation, use of guideline-directed therapies, in-hospital procedures, complications, readmission and mortality (inpatient, 30-day, and 1-year), and total number of AMI hospitalizations; analyze the associations between the level of monthly income (> 500 US$ *vs*. ≤ 500 US$) with clinical features, SES, and management of AMI; assess key performance indicators in the diagnostic work-up, evidence-based therapies, and cardiac procedures at admission and during follow-up; and assess the independent predictors of short- and long-term mortality and recurrent AMI hospitalization.

## Methods

### Study population

Consecutive patients hospitalized with type 1 AMI (STEMI and NSTEMI) defined according to the ESC Guidelines and ≥18 years of age were included in the registry. We excluded patients who had AMI due to an imbalance of the oxygen supply and demand, AMI resulting in death but without availability of biomarkers, or AMI related to percutaneous intervention (PCI) or coronary artery bypass grafting CABG (i.e., AMI types 2, 3, 4, or 5).

### Data collection

Baseline data were collected at hospital admission and included demographic characteristics, SES, education level, health care coverage, CVRs, clinical presentation, laboratory investigations, including cardiac troponin and high-sensitivity troponin, cardiac procedures, and treatments.

Patients with a previous history of coronary artery disease (CAD) were documented by coronary angiography, myocardial infarction (MI), or coronary revascularization. Diabetic patients had a confirmed diagnosis of diabetes mellitus written on their chart or were on diabetic medications. History of hypertension was collected from the patient’s chart or use of hypertensive medications. History of dyslipidemia was considered if written on the patient’s chart or demonstrated by electrocardiogram (ECG). History of atrial fibrillation (AF) was defined as previously documented AF, either paroxysmal or chronic, with or without oral anticoagulant agents or peripheral emboli. AF that developed in-hospital course was defined as AF requiring therapy: electrical or pharmacological cardioversion. Chronic kidney disease/dialysis was noted if GFR < 60 mL/min/1.73 m^2^ for 3 months or more, with or without kidney damage (NKF/KDOQI eGFR definition from MDRD equation) or the patient was on dialysis. If no GFR was available, serum creatinine > 177 mmol/L or 2 mg/dL was marked as chronic kidney disease (TIMI study group). Laboratory investigations and diagnostics were retrieved from the central hospital lab database. ECG and echocardiography data were collected from the cardiac non-invasive laboratory. Data on cardiac procedures, such as PCI, CABG, implantable cardioverter defibrillator (ICD), and cardiac resynchronization therapy (CRT), were collected from the catheterization laboratory and operating room records.

Information on the in-hospital course, cardiac procedures, and guideline-directed medical therapy was collected at admission, upon discharge, and at 1 year. Follow-up data on mortality and recurrent AMI hospitalization at 30 days and 1 year were collected from online case report forms (CRFs).

### Study design

The full study design has been described elsewhere [5]. Briefly, the study was divided into three phases. First, the pilot phase aimed to identify the logistic challenges and test the feasibility of completing the CRFs in ‘real-life’ practice. Next, in phase I, the results of which are reported in the present study, we measured the baseline clinical features, SES, and case management practices of patients admitted with AMI. We also assessed in-hospital outcomes, short- and long-term all-cause mortality rates, and rates of recurrent AMI hospitalization to discover knowledge–care gaps early in the study for future improvement of clinical practice. Finally, phase II will measure the same variables at a later time point to assess the effectiveness of this initiative in improving the quality of care.

### Study organization

Throughout the hospital stay of each patient, a CRF with data variables of standard definitions was completed online by dedicated research assistants and physicians. All CRFs were verified by a cardiologist and sent to the principal coordinating center, where the forms were checked for incomplete data and errors before submission for final analysis.

### Statistical analysis

Categorical data were summarized as absolute numbers and percentages, whereas continuous data were summarized as means and standard deviations (SDs) or medians and interquartile ranges (IQRs). Comparisons were performed between different groups using chi-squared or the Fisher’s exact test for categorical variables, and the student t-test or Mann Whitney U test for continuous data. The normality of the continuous variables was tested using Shapiro–Wilk and Kolmogorov–Smirnov tests. Univariate and multiple logistic regression models were used to identify univariate and multivariate risk factors for mortality and re-admission. All statistical analyses were performed using SAS version 9.2 (SAS Institute, Inc, Cary, NC) and R software (R Foundation for Statistical Computing, Vienna, Austria). A 2-sided P value < 0.05 was considered statistically significant.

### Ethics statement

Ethics approval was obtained from the Institutional Review Boards (IRBs) of the participating countries according to their relevant national regulations and laws. Saudi Arabia, King Saud University, College of Medicine IRB, OHRP No. IORG0006829. Qatar, Doha, HMC-IRB Registration: SCH-HMC-020-2015. Algeria, Hopital Mustapha Place du Premier Mai- Alger IRB. Kuwait, Ministry of Health, Secretary for Planning and Quality Committee. Yemen, Al Thawra Hospital Ethics Committee. Iraq, Baghdad Health Department, Ibn Naphis Hospital IRB. Egypt, Alexandria, International Cardiac Center IRB. Tunisia, Ministry of Health, Sahloul Hospital of Sousse. UAE, Hammoud Hospital IRB. Sudan, Sudan Heart Center IRB. Jordan, Istishari Hospital IRB. Morocco, Centre Hospitalo–Universitaire Ibn Sina IRB. Bahrain, BDF Royal Medical Services IRB. Oman, Ministry of Health, Directorate General of Planning and Studies.

## Results

Between April 2019 and 28 June 2020, 1377 patients with AMI (79.1% males) from 16 Arabian countries were recruited from a total of 37 hospitals (23 Cath hospitals vs. 14 non-Cath hospitals). The mean age (± SD) was 58 ± 12 years; 729 (53%) had STEMI and 648 (47%) had NSTEMI. Overall, the prevalence of CVRs was high; 48% of the cohort had diabetes mellitus, 57% had hypertension, 41.6% had hypercholesterolemia, and 51.6% were either current or ex-smokers. Upon presentation, 19.7% of patients were transferred to the emergency department in a hospital ambulance and 9.8% were transferred by the Emergency Medical Service (EMS). Compared to patients with STEMI, those with NSTEMI were more likely to have history of angina, myocardial infarction, PCI, CABG, heart failure, chronic renal failure, diabetes, hypertension, or hypercholesterolemia. Guideline-recommended treatments were given at high rates upon hospital admission: 99.6% of patients received acetylsalicylic acid (ASA), 88.9% received statins, 79.6% received beta-blockers, and 62.4% received ACE-I/ARBs. Interestingly, only 11.8% received ticagrelor, whereas 84.5% received clopidogrel. The overall use of guideline-recommended treatments remained high upon discharge: 99.1% received ASA, 89.3% received beta-blockers, 71% received ACE-I/ARBs, and 89.7% received statins. The use of ticagrelor increased, overall, to 12.7% at hospital discharge, and it was given significantly more frequently to patients with STEMI (15.7%) than those with NSTEMI (9.4%, p<0.001). In the STEMI group, 49.8% of patients underwent a primary PCI, 36.2% thrombolysis, and 13.8% received no acute reperfusion therapy. Door-to-balloon times <90 min was achieved in nearly two-thirds of patients, whereas door-to-needle time <30 min and door-to-ECG time <10 min was only achieved in approximately half of the patient cohort. Total ischemic time for primary PCI median (IQR) was 195(465) minutes and for thrombolytics 180 (440) with no significant difference between high vs low-income groups S1 Table, the overall length of stay (LOS) was 5.25 days with no significant difference between patients presenting with STEMI vs. NSTEMI. Nonetheless, length of stay was higher when comparing low income vs. high income groups (5.91(8.45) vs. 4.2(4.29) P<0.001 S2 Table. In-hospital mortality was 2.4% and occurred more frequently in the STEMI group than the NSTEMI group (3.3% vs. 1.4%; P = 0.021) Table 1. The use of radial access was noted almost half of the time in both STEMI and NSTEMI (49% and 56%, respectively).

**Table 1:** Baseline characteristics, clinical presentation, medications, outcomes and cardiac procedures in patients with acute myocardial infarction.

	STEMI N = 729(52.94%)	NSTEMI N = 648(47.06%)	Total N = 1377	P-value
**Age**	56.67 ± 11.74	59.67 ± 12.02	58.08 ± 11.97	< .001
**Male**	621 (85.19%)	468 (72.22%)	1089 (79.08%)	< .001
**Female**	108 (14.81%)	180 (27.78%)	288 (20.92%)	
**History of angina**	135 (18.52%)	207 (31.94%)	342 (24.84%)	< .001
**History of MI**	75 (10.29%)	134 (20.68%)	209 (15.18%)	< .001
**History of PCI**	83 (11.39%)	138 (21.30%)	221 (16.05%)	< .001
**History of CABG**	9 (1.23%)	41 (6.33%)	50 (3.63%)	< .001
**History of heart failure**	23 (3.16%)	73 (11.27%)	96 (6.97%)	< .001
**History of stroke**	25 (3.43%)	45 (6.94%)	70 (5.08%)	0.003
**History of chronic renal failure**	20 (2.74%)	61 (9.41%)	81 (5.88%)	< .001
**DM**	301 (41.29%)	361 (55.71%)	662 (48.08%)	< .001
**HTN**	359 (49.25%)	427 (65.90%)	786 (57.08%)	< .001
**Hypercholesterolemia**	286 (39.23%)	283 (43.67%)	569 (41.32%)	0.095
**Current or ex-smoking**	429 (58.85%)	282 (43.52%)	711 (51.63%)	< .001
**Transferred to ED by Ambulance**	168 (23.05%)	104 (16.05%)	272 (19.75%)	0.001
**Transferred by EMS**	84 (50.00%)	51 (49.04%)	135 (49.63%)	0.878
**HR > 100bpm**	107 (14.68%)	89 (13.73%)	196 (14.23%)	0.617
**SBP< 90mmgh**	28 (3.84%)	8 (1.23%)	36 (2.61%)	0.002
**CHF Killip Class**				0.006
**(No CHF)**	581 (79.70%)	514 (79.32%)	1095 (79.52%)	
**(Rales and/or Jugular venous distension)**	101 (13.85%)	92 (14.20%)	193 (14.02%)	
**pulmonary edema**	27 (3.70%)	38 (5.86%)	65 (4.72%)	
**Cardiogenic shock**	20 (2.74%)	4 (0.62%)	24 (1.74%)	
**Type of troponin available**				< .001
**cTn**	370 (50.75%)	213 (32.87%)	583 (42.34%)	
**hsTn**	359 (49.25%)	435 (67.13%)	794 (57.66%)	
**Type of (hsTn)**				< .001
**hsTnT**	185 (51.53%)	305 (70.11%)	490 (61.71%)	
**hsTnI**	174 (48.47%)	130 (29.89%)	304 (38.29%)	
**Choose one BNP: NT-pro BNP**				< .001
**BNP**	95 (65.97%)	70 (39.55%)	165 (51.40%)	
**NT-pro BNP (optional)**	49 (34.03%)	107 (60.45%)	156 (48.60%)	
**Echo**	648 (88.89%)	593 (91.51%)	1241 (90.12%)	0.103
**Echo-Options**				< .001
**Normal LV systolic function (EF >50%)**	245 (37.81%)	310 (52.28%)	555 (44.72%)	
**Mild LV systolic dysfunction (EF 40–50%)**	273 (42.13%)	187 (31.53%)	460 (37.07%)	
**Moderate LV systolic dysfunction (EF 30–40%)**	103 (15.90%)	75 (12.65%)	178 (14.34%)	
**Severe LV systolic dysfunction (EF <30%)**	27 (4.17%)	21 (3.54%)	48 (3.87%)	
**Elective coronary angiogram**	99 (19.60%)	122 (29.83%)	221 (24.18%)	< .001
**Elective PCI**	70 (9.60%)	87 (13.43%)	157 (11.40%)	0.026
**CABG**	19 (3.73%)	32 (7.80%)	51 (5.55%)	0.007
**24 Hrs Medications**				
**Aspirin**	727 (99.73%)	644 (99.38%)	1371 (99.56%)	0.335
**Clopidogrel**	602 (82.58%)	564 (87.04%)	1166 (84.68%)	0.022
**Prasugrel**	20 (2.74%)	10 (1.54%)	30 (2.18%)	0.128
**Ticagrelor**	110 (15.09%)	53 (8.18%)	163 (11.84%)	< .001
**Beta Blockers**	567 (77.78%)	530 (81.79%)	1097 (79.67%)	0.065
**ACEI or ARB**	483 (66.26%)	376 (58.02%)	859 (62.38%)	0.002
**Statins**	610 (83.68%)	614 (94.75%)	1224 (88.89%)	< .001
**Aldostr Spironol**	61 (8.37%)	51 (7.87%)	112 (8.13%)	0.736
**Heparins**	606 (83.13%)	547 (84.41%)	1153 (83.73%)	0.519
**GP2b3**	91 (12.48%)	28 (4.32%)	119 (8.64%)	< .001
**Bivalirudin**	11 (1.51%)	12 (1.85%)	23 (1.67%)	0.620
**Insulin**	244 (33.47%)	273 (42.13%)	517 (37.55%)	< .001
**OH**	45 (6.17%)	93 (14.35%)	138 (10.02%)	< .001
**Discharge Medications**				
**Aspirin**	702 (99.57%)	630 (98.59%)	1332 (99.11%)	0.056
**Clopidogrel**	572 (81.13%)	524 (82.00%)	1096 (81.55%)	0.682
**Prasugrel**	19 (2.70%)	12 (1.88%)	31 (2.31%)	0.319
**Ticagrelor**	111 (15.74%)	60 (9.39%)	171 (12.72%)	< .001
**Beta Blockers**	629 (89.22%)	572 (89.51%)	1201 (89.36%)	0.861
**ACEI or ARB**	550 (78.01%)	406 (63.54%)	956 (71.13%)	< .001
**Statins**	591 (83.83%)	615 (96.24%)	1206 (89.73%)	< .001
**MRA**	169 (23.97%)	76 (11.89%)	245 (18.23%)	< .001
**Oral Anticoagulants**	113 (16.03%)	77 (12.05%)	190 (14.14%)	0.037
**Insulin**	123 (17.45%)	156 (24.41%)	279 (20.76%)	0.002
**OH**	187 (26.52%)	214 (33.49%)	401 (29.84%)	0.005
**Recurrent ischemia**	51 (7.00%)	79 (12.19%)	130 (9.44%)	< .001
**Recurrent MI**	13 (1.78%)	15 (2.31%)	28 (2.03%)	0.485
**Atrial Fibrillation Flutter**	39 (5.35%)	52 (8.02%)	91 (6.61%)	0.046
**Heart Failure**	113 (15.50%)	85 (13.12%)	198 (14.38%)	0.208
**Cardiogenic Shock**	36 (4.94%)	12 (1.85%)	48 (3.49%)	0.002
**VTVF arrest**	45 (6.17%)	16 (2.47%)	61 (4.43%)	< .001
**IABP**	9 (1.23%)	14 (2.16%)	23 (1.67%)	0.181
**Stroke**	2 (0.27%)	7 (1.08%)	9 (0.65%)	0.064
**Major bleeding**	8 (1.10%)	6 (0.93%)	14 (1.02%)	0.752
**Cardiac Tamponade**	0 (0.00%)	1 (0.15%)	1 (0.07%)	0.289
**Stent thrombosis**	5 (0.69%)	0	5 (0.36%)	0.111
**Mortality**	24 (3.29%)	9 (1.39%)	33 (2.40%)	0.021

Abbreviations: STEMI: ST-elevation myocardial infarction, NSTEMI: non-ST-elevation myocardial infarction, PCI: percutaneous coronary intervention, CABG: coronary artery bypass graft, LV: left ventricular, EF: ejection fraction, IABP Intra-Aortic Balloon Pump Balloon bump. VTVF ventricular tachycardia and fibrillation, OH: oral hypoglycemic medication, MRA: Mineralocorticoid receptor antagonists, ACEI or ARB: angiotensin converting enzyme inhibitor and angiotensin receptor blockers, GP2b3: Glycoprotein IIb/IIIa inhibitors.

A review of SES revealed that almost one-quarter of the population had only primary education and less than one-third had secondary education; 14.5% had no education at all, and only 2% had a post-graduate degree. One-third of the population had medical insurance and 72.1% had free governmental medical care. Almost half off the population had a net income < $500/month, one-third had income between $500-2000/month, and almost one-third of the population had difficulty paying their bills Table 2.

**Table 2:** Socioeconomic status of acute myocardial infarction patients.

	STEMI	NSTEMI	Total	P-value
**Education**				0.018
**None**	93/729 (12.76%)	107/648 (16.51%)	200/1377 (14.52%)	
**Primary**	174/729 (23.87%)	172/648 (26.54%)	346/1377 (25.13%)	
**Secondary/high school/Diploma (2years after high school)**	216/729 (29.63%)	179/648 (27.62%)	395/1377 (28.69%)	
**Trade school/vocational school**	71/729 (9.74%)	62/648 (9.57%)	133/1377 (9.66%)	
**College/university**	165/729 (22.63%)	110/648 (16.98%)	275/1377 (19.97%)	
**Post-graduation degree. e.g., PHD, Master, Diploma**	10/729 (1.37%)	18/648 (2.78%)	28/1377 (2.03%)	
**Income**				0.029
**< 200$**	207/729 (28.40%)	177/648 (27.31%)	384/1377 (27.89%)	
**200–500$**	259/729 (35.53%)	210/648 (32.41%)	469/1377 (34.06%)	
**500–2000$**	202/729 (27.71%)	172/648 (26.54%)	374/1377 (27.16%)	
**2000–4000$**	35/729 (4.80%)	56/648 (8.64%)	91/1377 (6.61%)	
**>4000$**	26/729 (3.57%)	33/648 (5.09%)	59/1377 (4.28%)	
**Total number of family members that you are responsible for financially?**	4.00 (3.00)	3.00 (4.00)	4.00 (3.00)	< .001
**Difficulties with paying bills**	257/729 (35.25%)	177/648 (27.31%)	434/1377 (31.52%)	0.002
**how often did it happen in the last year?**				0.858
**All the time**	37/257 (14.40%)	28/177 (15.82%)	65/434 (14.98%)	
**Often**	88/257 (34.24%)	65/177 (36.72%)	153/434 (35.25%)	
**Sometimes**	126/257 (49.03%)	81/177 (45.76%)	207/434 (47.70%)	
**Rarely**	6/257 (2.33%)	3/177 (1.69%)	9/434 (2.07%)	
**Are you covered by a private company medical insurance?**				
**Medical insurance**	218/729 (29.90%)	183/648 (28.24%)	401/1377 (29.12%)	0.498
**Free governmental medical care**	353/511 (69.08%)	351/465 (75.48%)	704/976 (72.13%)	0.026
**Difficulties medical care expenses**	91/158 (57.59%)	58/114 (50.88%)	149/272 (54.78%)	0.272
**How often?**				0.258
**All the time**	14/91 (15.38%)	13/58 (22.41%)	27/149 (18.12%)	
**Often**	27/91 (29.67%)	17/58 (29.31%)	44/149 (29.53%)	
**Sometimes**	39/91 (42.86%)	26/58 (44.83%)	65/149 (43.62%)	
**Rarely**	11/91 (12.09%)	2/58 (3.45%)	13/149 (8.72%)	
**Please indicate which group best describes your current occupation?**				0.026
**Self-employed (as Independent, or have own business)**	160/729 (21.95%)	121/648 (18.67%)	281/1377 (20.41%)	
**Employee (as salesperson, director, accountant)**	264/729 (36.21%)	226/648 (34.88%)	490/1377 (35.58%)	
**Retired**	160/729 (21.95%)	129/648 (19.91%)	289/1377 (20.99%)	
**Unemployed (as housewife, househusband)**	145/729 (19.89%)	172/648 (26.54%)	317/1377 (23.02%)	

Comparing patients who had low income (≤$500/month) vs. high income (>$500/month), there was no significant difference in the prevalence of CVRs, such as hypertension, diabetes mellitus, and hypercholesterolemia, except for smoking, which occurred less frequently in the lower income group (49.1% vs. 55.7%; P = 0.017). Furthermore, primary PCI was performed less frequently in the lower income group (26.3% vs. 54.7%; P<0.001), but with no difference in the group that achieved a door-to-balloon time <90 min. When comparing outcomes between the low income and high-income populations, there was no difference in in-hospital mortality, stroke, or heart failure, but recurrent ischemia occurred more frequently in the low-income group (10.9% vs. 7%; P = 0.018) Table 3.

**Table 3:** Income characteristics and outcomes of acute myocardial infarction patients.

	< = 500 $ (n = 853)	> 500 $(n = 524)	Total(n = 1377)	P-value
**Age**	59.08 ± 11.93	56.47 ± 11.86	58.08 ± 11.97	< .001
**Gender**				
**Male**	625 (73.27%)	464 (88.55%)	1089 (79.08%)	< .001
**STEMI**	466 (54.63%)	263 (50.19%)	729 (52.94%)	0.109
**GCC**	222 (26.03%)	287 (54.77%)	509 (36.96%)	< .001
**Education**				< .001
**Non-primary and Secondary**	441 (51.70%)	105 (20.04%)	546 (39.65%)	
**Others**	412 (48.30%)	419 (79.96%)	831 (60.35%)	
**HTN**	495 (58.03%)	291 (55.53%)	786 (57.08%)	0.364
**DM**	419 (49.12%)	243 (46.37%)	662 (48.08%)	0.322
**Current or ex-smoking**	419 (49.12%)	292 (55.73%)	711 (51.63%)	0.017
**Hypercholesterolemia**	338 (39.62%)	231 (44.08%)	569 (41.32%)	0.103
**History of MI Angina**	237 (27.78%)	170 (32.44%)	407 (29.56%)	0.066
**History of PCI**	138 (16.18%)	83 (15.84%)	221 (16.05%)	0.868
**History of CABG**	31 (3.63%)	19 (3.63%)	50 (3.63%)	0.994
**History of heart failure**	59 (6.92%)	37 (7.06%)	96 (6.97%)	0.919
**History of stroke**	51 (5.98%)	19 (3.63%)	70 (5.08%)	0.054
**History of chronic renal failure**	45 (5.28%)	36 (6.87%)	81 (5.88%)	0.222
**HR > 100 bpm**	115 (13.48%)	81 (15.46%)	196 (14.23%)	0.308
**SBP < 90 mmgH**	24 (2.81%)	12 (2.29%)	36 (2.61%)	0.554
**CHFKILLIPCLASSS**				0.272
**Killip Class III and IV**	60 (7.03%)	29 (5.53%)	89 (6.46%)	
**Killip Class I and II**	793 (92.97%)	495 (94.47%)	1288 (93.54%)	
**ECHO OPTIONS**				0.445
**Moderate and Severe**	135 (17.56%)	91 (19.28%)	226 (18.21%)	
**Normal and Moderate**	634 (82.44%)	381 (80.72%)	1015 (81.79%)	
**Elective Emergency PCI**	153 (17.94%)	89 (16.98%)	242 (17.57%)	0.652
**Primary PCI done**	123 (26.39%)	144 (54.75%)	267 (36.63%)	< .001
**Door to balloon < 90 Minutes**	85 (69.11%)	105 (72.92%)	190 (71.16%)	0.493
**Medications At Hospital Discharge**				
**Anti-platelets**	822 (99.16%)	513 (99.61%)	1335 (99.33%)	0.319
**Beta Blockers**	737 (88.90%)	464 (90.10%)	1201 (89.36%)	0.490
**ACEI or ARB**	606 (73.10%)	350 (67.96%)	956 (71.13%)	0.043
**Statins**	707 (85.28%)	499 (96.89%)	1206 (89.73%)	< .001
**Aldost Spironolactone**	172 (20.75%)	73 (14.17%)	245 (18.23%)	0.002
**Major in-hospital Outcomes**				
**Recurrent ischemia**	93 (10.90%)	37 (7.06%)	130 (9.44%)	0.018
**Recurrent MI**	16 (1.88%)	12 (2.29%)	28 (2.03%)	0.597
**Atrial Fibrillation Flutter**	66 (7.74%)	25 (4.77%)	91 (6.61%)	0.031
**Heart Failure**	125 (14.65%)	73 (13.93%)	198 (14.38%)	0.711
**Cardiogenic Shock**	31 (3.63%)	17 (3.24%)	48 (3.49%)	0.702
**VT/VF arrest**	41 (4.81%)	20 (3.82%)	61 (4.43%)	0.386
**IABP**	20 (2.34%)	3 (0.57%)	23 (1.67%)	0.013
**Stroke**	7 (0.82%)	2 (0.38%)	9 (0.65%)	0.326
**Dead**	24 (2.81%)	9 (1.72%)	33 (2.40%)	0.197

Values are the number of patients (%), PCI: Percutaneous coronary intervention, CABG: Coronary artery bypass surgery, HTN: hypertension, MI: myocardial infarction, STEMI: ST-elevation myocardial infarction, VF: Ventricular fibrillation, VT: Ventricular Tachycardia, IABP: Intra-Aortic Balloon Pump, ARBs: Angiotensin receptor blockers, ACEIs: Angiotensin-converting enzyme inhibitors, DM: Diabetes mellitus, GCC: The Gulf Cooperation Council.

When comparing Cath vs. non-Cath hospitals, primary PCI was performed more frequently in Cath hospitals (52.8% vs. 42.8%; P = 0.013), whereas thrombolytic therapy was preformed less frequently in Cath hospitals (32.4% vs. 44.6%; P = 0.002) Table 4.

**Table 4:** Revascularization for STEMI patients in Cath vs. non-Cath hospitals.

	Cath-Lab Hospital N = 505(69.27%)	Non-Cath-Lab Hospital N = 224(30.73%)	Total N = 729	P-value
**thrombolytic**	164 (32.48%)	100 (44.64%)	264 (36.21%)	0.002
**Clinical Signs of Reperfusion**	69 (13.66%)	63 (28.13%)	132 (18.11%)	< .001
**Rescue Cath PCI**	63 (12.48%)	0 (0.00%)	63 (8.64%)	< .001
**Pharmaco invasive**	54 (10.69%)	6 (2.68%)	60 (8.23%)	< .001
**Primary PCI done/transferred**	267 (52.87%)	96 (42.86%)	363 (49.79%)	0.013
**No reperfusion therapy**	73 (14.46%)	28 (12.50%)	101 (13.85%)	0.481
**Symptom to ER Minutes**	224.0 (575.0)	61.00 (114.5)	154.0 (390.0)	< .001
**Door to ECG Minutes**	10.00 (10.00)	10.00 (10.00)	10.00 (10.00)	0.224
**Door to ECG < 10 Minutes**	247 (48.91%)	101 (45.09%)	348 (47.74%)	0.341
**Door to needle Minutes**	60.00 (83.00)	30.00 (25.00)	45.00 (48.00)	0.177
**Door to needle 30Min**	16 (13.45%)	41 (46.07%)	57 (27.40%)	< .001

PCI: Percutaneous coronary intervention, ER: Emergency room, ECG: electrocardiogram.

Interestingly, ticagrelor was used more often than clopidogrel in Cath hospitals (ticagrelor: 18.5% vs. 1.3% in non-Cath; P<0.001; clopidogrel: 74.6% vs. 95.1%; P<0.001). There was no difference in in-hospital outcome (mortality, MI, and stroke) between the two groups, though the use of intra-aortic-balloon-pump (IABP) was noted to be higher in Cath hospitals, along with the occurrence of major bleeding and heart failure (16.4% vs. 10.3%; P = 0.003) Table 5.

**Table 5:** Overall Cath vs, non-Cath hospital.

	Cath-Lab Hospital N = 914(66.38%)	Non-Cath-Lab Hospital N = 463(33.62%)	Total N = 1377	P-value
**Type of troponin available**				< .001
**cTn**	435 (47.59%)	148 (31.97%)	583 (42.34%)	
**hsTn**	479 (52.41%)	315 (68.03%)	794 (57.66%)	
**Type of (hsTn)**				< .001
**hsTnT**	266 (55.53%)	224 (71.11%)	490 (61.71%)	
**hsTnI**	213 (44.47%)	91 (28.89%)	304 (38.29%)	
**Echo**	816 (89.28%)	425 (91.79%)	1241 (90.12%)	0.140
**Echo-Options**				< .001
**Normal LV systolic function (EF >50%)**	336 (41.18%)	219 (51.53%)	555 (44.72%)	
**Mild LV systolic dysfunction (EF 40–50%)**	307 (37.62%)	153 (36.00%)	460 (37.07%)	
**Moderate LV systolic dysfunction (EF 30–40%)**	142 (17.40%)	36 (8.47%)	178 (14.34%)	
**Severe LV systolic dysfunction (EF <30%)**	31 (3.80%)	17 (4.00%)	48 (3.87%)	
**Discharge Medications**				
**Aspirin**	882 (99.10%)	450 (99.12%)	1332 (99.11%)	0.974
**Clopidogrel**	664 (74.61%)	432 (95.15%)	1096 (81.55%)	< .001
**Prasugrel**	28 (3.15%)	3 (0.66%)	31 (2.31%)	0.004
**Ticagrelor**	165 (18.54%)	6 (1.32%)	171 (12.72%)	< .001
**Beta Blockers**	786 (88.31%)	415 (91.41%)	1201 (89.36%)	0.082
**ACEI or ARB**	609 (68.43%)	347 (76.43%)	956 (71.13%)	0.002
**Statins**	858 (96.40%)	348 (76.65%)	1206 (89.73%)	< .001
**Aldost Spironolactone**	164 (18.43%)	81 (17.84%)	245 (18.23%)	0.793
**Oral Anticoagulants**	106 (11.91%)	84 (18.50%)	190 (14.14%)	0.001
**Insulin**	196 (22.02%)	83 (18.28%)	279 (20.76%)	0.110
**OH**	284 (31.91%)	117 (25.77%)	401 (29.84%)	0.020
**Recurrent ischemia**	90 (9.85%)	40 (8.64%)	130 (9.44%)	0.469
**Recurrent MI**	16 (1.75%)	12 (2.59%)	28 (2.03%)	0.296
**Atrial Fibrillation Flutter**	66 (7.22%)	25 (5.40%)	91 (6.61%)	0.199
**Heart Failure**	150 (16.41%)	48 (10.37%)	198 (14.38%)	0.003
**Cardiogenic Shock**	36 (3.94%)	12 (2.59%)	48 (3.49%)	0.198
**VT/VF arrest**	50 (5.47%)	11 (2.38%)	61 (4.43%)	0.008
**IABP**	23 (2.52%)	0 (0.00%)	23 (1.67%)	< .001
**Stroke**	6 (0.66%)	3 (0.65%)	9 (0.65%)	0.985
**Major bleeding**	14 (1.53%)	0 (0.00%)	14 (1.02%)	0.007
**Stent thrombosis**	5 (0.55%)	1 (0.22%)	6 (0.44%)	0.378
**Mortality**	24 (2.63%)	9 (1.94%)	33 (2.40%)	0.434

Abbreviations: cTn: Conventional/ cardiac Troponin, hsTn: High-sensitive Troponin T, OH: Oral Hypoglycemic VF: Ventricular fibrillation, VT: Ventricular Tachycardia IABP: Intra-Aortic Balloon Pump, LV: left ventricular, EF: ejection fraction.Regarding follow-up, re-admission was noted to be 9% at 1 month and 30% at 1 year, whereas mortality was 0.7% at 1 month and 4.7% at 1 year.

Exploring key performance indicators, we found the following. Half of the population had STEMI, of which only half had primary PCI. Two-thirds of those who had primary PCI achieved door-to-balloon <90 min. Antiplatelet therapy uptake upon discharge and follow-up was high in all countries. Interestingly, the use off ticagrelor was higher in Saudi Arabia than other countries; on discharge, 61% of patients in Saudi Arabia were on ticagrelor and 40% on clopidogrel. Furthermore, there was high use of evidence-based medicine therapy on discharge and follow-up in all countries except Sudan and Palestine. For NSTEMI, the rate of revascularization varied significantly among countries: Yemen, Sudan, and Palestine had almost minimal revascularization. For STEMI, (72%) of patients in Morocco had no reperfusion therapy, but there was still high use of thrombolytic therapy in UAE (90% of patients) S3 Table.

## Discussion

The PEACE MENA registry provides data on the current management of AMI in the majority of Arabian countries in the MENA region. The main findings were that the AMI patients presented at a younger age compared to international registries and had a high burden of CVRs with low use of emergency medical services. The unique feature of phase 1 of the PEACE MENA registry is that it evaluated the SES in patients presenting with AMI in the MENA region.

In our study, we found different distributions of acute coronary syndrome (ACS) types in the PEACE MENA cohort compared to previous registries. We found higher prevalence of STEMI (53%) than NSTEMI (47%). In ACCESS, the prevalence was 46% and 54%, respectively [6], but were 41.5% and 68.5% in SPACE and 65% and 35% in STARS [7, 8]. This may be explained by the nature of the centers participating in PEACE MENA being tertiary care centers with Cath-lab capabilities, recruiting more patients with STEMI. In addition, as was shown before in the region we found a relatively younger age of patients presenting with ACS (58) with very high rates of CVRs especially DM as compared to other registries in the western world such as the Fast MI and CRUSADE registries [9, 10]. Furthermore, we noted a low transfer rate of STEMI patients to the emergency department via ambulance services (23%) or EMS (11.5%). This finding was noted in our pilot study and the Gulf RACE 3Ps registry [11]. This low use of emergency services contrasts with other registries, such the French Fast AMI registry in which 62% of STEMI patients were transferred by EMS to the hospital [9].

This study was the first to investigate the rate of radial access in patients with AMI in the MENA region, almost half of the time in both STEMI and NSTEMI. Radial access is the preferred choice because it is associated with lower mortality and complication rates across the spectrum of patients with CAD [12]. In Indonesia, radial access was reported to be used in 74.5% of the cases in nine participating centers [13]. Though this high uptake was not found in other registries, such as the SWEDEHEART registry, in which only 54.2% has radial access [14], in Germany the Quik registry showed a steady increase in the use radial access (from 13% to 49%) over the period from 2012 to 2018 [15]. In India, the Kerala Primary Percutaneous Coronary Intervention Registry demonstrated variability in use of radial access according to volume of the hospital (60–70%) [16].

SES is measured by multiple different parameters, including education, wealth, and occupation, and can be aggregated to define neighborhood or area-level SES [17]. Prior systematic reviews and meta-analyses have demonstrated the clear inverse relationship between CVD and CVRs and the level of education. Furthermore, low education level was associated with higher prevalence of smoking, obesity, hypertension, diabetes, and sedentary lifestyle [18–22]. This is clearly detected in the MENA region, where a high prevalence of CVRs and CVD with low levels of education (40% of the population in our study had no formal education or only primary education). Macken Bach et al. showed in a comparative study of the USA and 11 high-income European countries that CVD mortality is higher among people with lower occupational position [23]. In the MENA region, this factor has been a contributor to CVD because 23% of the population in our study were unemployed.

Low income and its relationship with CVRs and CVEs parallels that of education and occupation. Khaing et al. described, in a meta-analysis of four cohorts from Asia, Europe, and USA, an increased risk of CVRs and CVEs with low income and education [24]. This relationship is better understood in high-income countries than low-income countries. The evidence is scarcer in low-income countries, as demonstrated in small studies from India and Latin America [25, 26]. Further evidence is required in this area, especially in the MENA region; 28% of the population in our study had a low income and developed more recurrent ischemia and underwent less primary PCI hence likely explain the longer duration of hospital stay.

As mentioned earlier, the relationship between SES and CVD and CVD outcomes has been better studied in high-income countries than middle-income and low-income countries. Annika Rosengren et al. described the relationship between SES and CVD and outcomes was still apparent in middle- and low-income countries, with education being the strongest predictor. This gradient between SES and CVD and outcomes was also steepest in low-income countries [27].

For key performance indicators, we found high use of evidence-based medicine in all MENA regions, except areas with political instability and low SES, such as Sudan and Palestine. We also noted increased use of ticagrelor compared to prior registries in the region and the low use of primary PCI in STEMI patients, as well as a high number of patients with STEMI who did not receive any reperfusion. In contrast, in the Acute Coronary Syndrome STEMI registry of the EURObservational Research Programme and ACVC and EAPCI Association of the European Society of Cardiology [28], more than 70% of patients received PCI, 18% received thrombolytics, and 9% had no reperfusion. Such a variation was also noted among patients not receiving PCI or thrombolytics: 15.1% in the Middle East and 2.5% in Western Europe. This highlights the underutilization of primary PCI and increased proportion of patients who do not receive any form of reperfusion.

The limitations of this study include the inherent nature of observational studies leading to potential selection bias and the possibility of missing or incomplete data at the 1-year follow-up. Hospital enrollment was voluntary, with a lack of involvement of the outpatient population. Furthermore, the elapsing time during the COVID-19 pandemic may be a limitation. Some hospitals were directed to receive only coronavirus patients. The data collectors were obligated to stop patients’ data collection. This situation highly affected the recruitment from these hospitals (e.g., Kuwait and UAE). Moreover, because of the lockdown in many countries, the data collectors found it difficult to meet the patients in person in the clinic for follow-up. Because of the precautionary measures taken for coronavirus in many hospitals, in-person contact with the patients was minimized.

In conclusion, our registry has similar finding as prior registries in which patients with AMI present at a young age with a high burden of CVRs. We also found that most patients had low income and low education status, with large heterogeneity in key performance indicators of AMI management. This will help in understanding the deficiencies in heath care systems in the region and give rise to future research and quality improvement initiatives.

## Supporting information

S1 File Questionnaire-typeset39MIO. (DOCX)

S1 Table Total ischemic time for STEMI. (DOCX)

S2 Table Length of stay (LOS) for STEMI vs NSTEMI. (DOCX)

S3 Table Key performance indicators for AMI patients in the MENA region. (XLSX)
