# Supplementary material for: Clinical features, socioeconomic status, management, short and long-term outcomes of patients with acute myocardial infarction: Phase I results of PEACE MENA registry
Source: PLoS One. 2024 Jan 11;19(1):e0296056. doi: 10.1371/journal.pone.0296056 (PMC10783754; doi:10.1371/journal.pone.0296056)

S1 Table. Total ischemic time for STEMI.

| STEMI patients | Low income | High income | Total | P-value |
| --- | --- | --- | --- | --- |
| Primary PCI: Total ischemic time , median (IQR) | 185(599) | 210(339) | 195(465) | 0.191 |
| Thrombolytics: Total ischemic time , median (IQR) | 180(538) | 165(340) | 180(440) | 0.585 |


Supplement: S1 Table — (DOCX) [file pone.0296056.s002.docx]
