# Supplementary material for: Clinical features, socioeconomic status, management, short and long-term outcomes of patients with acute myocardial infarction: Phase I results of PEACE MENA registry
Source: PLoS One. 2024 Jan 11;19(1):e0296056. doi: 10.1371/journal.pone.0296056 (PMC10783754; doi:10.1371/journal.pone.0296056)
Supplement: S2 Table — (DOCX) [file pone.0296056.s003.docx]

S2 Table Length of stay (LOS) for STEMI vs NSTEMI.

|  | STEMI | NSTEMI | overall | P-value |
| --- | --- | --- | --- | --- |
| LOS | 5.02(6.79) | 5.52(7.61) | 5.25(7.19) | 0.202 |

Length of stay for Low income vs. high income.

|  | Low income | High income | overall | P-value |
| --- | --- | --- | --- | --- |
| LOS | 5.91(8.45) | 4.20(4.29) | 5.25(7.19) | <0.001 |
